# Assessment of injectable and cohesive nanohydroxyapatite composites for biological functions

**DOI:** 10.1007/s40204-014-0034-7

**Published:** 2014-12-17

**Authors:** Sai Santosh Babu Komakula, Snehal Raut, Nitin Pratap Verma, T. Avinash Raj, Mahesh J. Kumar, Arvind Sinha, Shashi Singh

**Affiliations:** 1 CSIR-Centre for Cellular and Molecular Biology, Uppal Road, Hyderabad, 500 007 India; 2 CSIR-National Metallurgical Laboratory, Jamshedpur, 831 007 India

**Keywords:** Biphasic apatite, Bioactivity, Osteointegrity, Bone drill injury, Histology

## Abstract

Pressing need for utilization of injectables/fillers in various forms of orthopaedic treatments/surgeries commands an equal demand for better graft material. Injectable bone graft material based on biomimetically synthesized nanohydroxyapatite was developed and subjected to ball milling for different times; three materials thus produced were evaluated for their biological properties. The three composites tested were found to have some difference in proliferation and differentiation on mesenchymal stem cells in cultures. In vivo studies were performed by implanting the graft materials with or without cells in the bone drill hole injury created in the femur of Wistar rats. Our studies show that the composites lead to well-healed injury site with normal histology without inflammation or fibrous tissue formation and bone deformity. This material needs to be tested on large animals for further ascertaining its applicability in clinical use.

## Introduction

Autologous bone grafts are most desirable for a bone injury that requires surgical intervention and grafting. Apart from availability, autologous bone grafts present difficulties in donor-site morbidity and contouring. Allogeneic bone grafts incur host of other problems like transmission of diseases, immune compatibility (Nandi et al. 2006; Schlickewei and Schlickewei 2006; Kokubo 2008). One option to overcome these problems is to use synthetic material that can be prepared in abundance, custom made to suit the requirement and properties of the implant site. Among these, the most successful and well tolerated is the calcium phosphate ceramics due to its good biocompatibility and bioactivity, and their use dates back to 19th century (Bohner 2000; Oh et al. 2006). Synthetic hydroxyapatite has found extensive use as a bone replacement material due to its chemical similarity to the apatites found in vertebrates. Most of the implant materials used in orthopaedics are prefabricated and hardened that require fitting sizes or carving around the implant to place it properly. One more option is to carve the graft to desired shape and size during the procedure.

Most of the time, it is the type of injury that decided the choice of graft, the material required and its form (as powder, solid three-dimensional graft; self-setting putty or a self-setting injectable bioceramic). Injectables are ideal for non-union or critical size injuries that fail to heal on their own like bone damage or disintegration caused by ageing, trauma. Use of injectable graft materials is also convenient where the bone contouring is must (Jackson and Yavuzer 2000; Verret et al. 2005), vertebral injuries (Bai et al. 1999; Hernández et al. 2009) and while opting for minimal invasive surgery. Various injectable materials like polymers, granules, and cements have been in use for filling gaps and contouring (Le Geros et al. 1982; Hu et al. 2010; Ślósarczyk et al. 2010; Tomoaia et al. 2011). Among polymers, methyl methacrylate is quite commonly used though it lacks properties of osteointegration and could result in necrosis and infection. These polymer-based materials also suffer from exothermic setting reactions (Vallo et al. 1999). Calcium phosphate (Ca–P) cements were introduced by Brown and Chow 1983 and these self-curing cements proved safe as they are made of mineral closer to natural bone matrix and show osteocompatibility and integration. The isothermic self-setting property of these materials helps in pre- or in situ moulding and can be used in a variety of settings. Ca–P based cements comprise of more than one component of calcium phosphate minerals that sets when injected in combination with water or sodium phosphate (Miyamoto et al. 1995; Kurashina et al. 1997; Fellah et al. 2006; Zhang et al. 2006). Although the setting reaction of Ca–P cement is also exothermic, the compositional modifications can minimize the increase in temperature, keeping it in a relatively safer zone to biological cells. Most of these injectables have low strength, are disintegrated easily in body fluids or water, and the long setting times in situ might lead to certain amount of wash out and non-integration. (Miyamoto et al. 1997).

Calcium phosphate ceramics including hydroxyapatite in pure form or in combination with tricalcium phosphates as bi- or triphasic calcium phosphates with better healing properties are quite popular for bone regeneration. Biphasic calcium phosphates exhibit osteoconductive properties of HA and osteoinductive nature of tricalcium phosphates making it a bioactive composite (Bohner 2000; Reddy et al. 2013).

Recently, a process has been developed to convert biomimetically synthesized biphasic nanoHA into injectable and self-setting, highly cohesive stable HA scaffolds, without involving the conventional ingredients of bone cement. The physicochemical characterization of the set cements, have been reported (Varma et al. 2012, Verma and Sinha 2013). In the present study, we have used these injectables to study the biocompatibility and bone healing properties using a bone injury model in Wistar rats.

## Materials and methods

### Synthesis and setting of nanocrystalline hydroxyapatite

Synthesis of injectable nanocrystalline HAP (Hydroxyapatite) powder was carried out following a Poly(vinyl alcohol) (PVA) matrix-mediated biomimetic synthesis (Sinha et al. 2003). In brief, an alkaline solution of calcium nitrate tetrahydrate was stirred vigorously into PVA solution and allowed to set as gel at 75 °C in hot air oven. Alkaline solution of diammonium hydrogen phosphate was added to the gels for precipitation and incubated for 48 h. The product was thoroughly washed, oven dried and sieved to collect particles below 250 MIC and labelled as N1. The N1 powder thus produced was further subjected to ball milling for 10 and 20 min and two composites N2 and N3 were produced (Varma et al. 2012, Verma and Sinha 2013) (Table 1). The composites were mixed with an optimum volume of neutral phosphate buffer maintaining a solid/liquid ratio of 1.08 g/ml and thoroughly mixed and filled in a syringe to fill up at the site of injury or casted by filling up in metallic moulds to produce pellet-shaped samples or coated as slurry on surface of cover slips. The cast material were immediately transferred to incubator at 35–37 °C and incubated for 20 min. The composite sets as solid structures in each case and is insoluble.

**Table 1:** Processing of HAP composites

Material	Processing
N1	NanoHAP powder
N2	NanoHAP powder after 10 min ball milling
N3	NanoHAP powder after 20 min ball milling

### Cell culture studies

Mesenchymal stem cells (MSC) isolated from human placenta maintained in the laboratory conditions as described in (Reddy et al. 2013) were used for assessing the in vitro biocompatibility with the ceramic.

The moulded pellets of the injectables were autoclaved before co-culturing with MSC in a Wheaton roller culture device at 5 rpm. 250,000 cells were suspended in Falcon tubes containing Iscove’s Modified Dulbecco’s Medium (IMDM) with the moulded hydroxyapatite pellets. The pellets were taken out after 6 and 12 h to show adherence of cells. The pellets were fixed in 2.5 % glutaraldehyde for 3 h, dehydrated through acetone series, passed through amyl acetate and dried in critical point drier. The dried samples were coated with gold and observed in Hitachi-S3400 N in SEM mode at 10 kV.

MSC derived from placenta were grown on coverslips coated with thin coats of the sterile injectable to assess their adherence, viability and growth. 25,000 cells were plated on coated coverslips and subjected to MTT assay after 24 and 120 h of plating. Cells plated on uncoated coverslips were used as control. After performing MTT, the supernatants were centrifuged to remove any particles before taking OD; Values obtained at 570 nm were used to assess the viability proliferation of cells. Results were expressed as percent of control.

### Expression of osteomarkers

RNA was isolated from cells grown on the three composites directly using Trizol reagent and converted to cDNA using superscript II. Glucose-6 phosphate (GAPDH), 18S ribosomal RNA and β-2 microglobulin (B2 M) served as internal controls. Real-time PCR was carried out for BMP2, BSP and osteocalcin genes (Table 2). The primers were custom synthesized by Bioserve India Ltd. and optimized using a crosswise combination matrix. A total of 100 ng cDNA was used for real-time PCR with SyBR Green as indicator using the Applied Biosystem (7900) HT fast real-time PCR system. Fold changes in gene expression were calculated by ΔΔCT method. Statistical analysis (two-way ANOVA) was carried out using GraphPad prism.

**Table 2:** Processing of HAP composites

Osteogenic markers		Sequence
BMP-2	F	AACGGACATTCGGTCCTTGC
	R	CGCAACTCGAACTCGCTCG
BSP	F	CTGGCACAGGGTATACAGCGTTAG
	R	ACTGGTGCCGTTTATGCCTTG
Osteocalcin	F	GGCAGCGAGGTAGTGAAGAG
	R	CTGGAGAGGAGCAGAACTGG
GAPDH	F	AGGGGTCTACATGGCAACTG
	R	CGACCACTTTGTCAAGCTCA
TBP	F	TGCACAGGAGCCAAGAGTGAA
	R	CACATCACAGCTCCCCACCA
18S RP	F	CCCAGTGCTCTGAATGTCAA
	R	AGTGGGAATCTCGTTCATCC
B2M	F	GGCTATCCAGCGTACTCCAA
	R	GATGAAACCCAGACACATAGCA

### Animal experiments

Selection of preclinical animal model to assess performance of an injectable bone substitute remains a challenge for want of a gold standard. We decided to use Wistar rats and create a drilled hole injury in femur to test the proof of principle. Skeletally mature 3-month-old female Wister rats of body weight 150–200 g from the animal house were used for the experiment. The animals were maintained at standard environmental conditions (temperature 22–25 °C, humidity 40–70 % with 12:12 dark/light photoperiod) approved by the Committee for the Purpose of Control and Supervision of Experiments on Animals (CPCSEA) where as all the experimental protocols were approved by IAEC, CCMB.

Animals were anesthetized with ketamine and xylazine with a dose of 40 mg and 5 mg/Kg body weight, respectively. Lateral and median aspect of the femur was cleaned. An incision was made to cut open the skin on the dorsal aspect above the femur. A careful incision was then made to cut open the biceps femoris muscles to expose femur bone. A paediatric bone driller was used to make a hole of 6 mm in the dorsal surface of femur without touching the bone marrow thus preventing any haemorrhage. Material + cell (TE constructs) were prepared with human placental MSC for each of the nanoceramic cements. N1, N2 and N3; the TE constructs were implanted in the drilled cavity of the rats [Group I—3 animals each]. In each TE construct, placental MSC (100,000 cells) were mixed with ceramic paste and filled into the drilled hole. In Group II [3 animals each], animals received acellular ceramic paste (N1, N2 and N3). The drilled femur of control animals was left unfilled and in one control, the animal received only cells. Ceramic was allowed to set before stitching the wound back. The muscle layer was stitched with simple interrupted suture using absorbable materials and skin was closed using non-absorbable suture materials. Povidone–iodine ointment was applied externally for 3 days. The animals were subjected to X-ray imaging. At the end of the experiment, the animals were killed by overdose of CO_2_. Detail necropsy was done and femur bone was collected and fixed in 10 % formalin. Fixed bone was decalcified with EDTA before embedding in paraffin wax. Fixed and paraffin-embedded bones were sectioned at 5 µm thickness, stained with haematoxylin and eosin following standard procedure and examined under light microscope.

### Results and discussion

The idea of an injectable bone graft material is to have an easy handy and sterile preparation procedure for clinicians to inject at desired location with minimal excision, a material having an easy flow for injectability, short setting time, and good compressive strength. The added advantages would be biocompatibility, with good bioactivity, low wash out/leaching, stability and a good porous structure. Our earlier papers (Varma et al. 2012; Verma and Sinha 2013) on the synthesis, setting and characterization of these graft materials have proved that the setting process is nonexothermic and these self-setting nanoHA-based cements are stable in water and blood. Further studies also showed that solid/liquid ratio can affect injectability, compressive strength and modulus of injectable nanoHA. Once the mechanical properties and suitability of bone graft mouldable materials was established; further studies were done to check its biofunctionality.

**Fig. 1:**
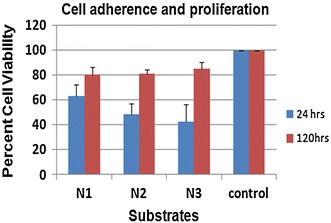
Cell adherence and proliferation studies on biocomposite material. Cells were grown on surfaces coated with N1, N2 and N3 and MTT assay was performed at 24 and 120 h to assess cell adherence and proliferation on these materials. Cell adherence was highest in N1 as observed at 24 h. Cell proliferation was seen in all composites to varying degree. The data are represented as percent of control

The preformed pellets/injectable hydroxyapatite-coated coverslips were autoclaved before plating placental-derived MSC. MTT assay was performed to check the viability and proliferation of cells. Direct seeding approach was used and the coated coverslips were layered with 25,000 cells and the assay was performed after 24 and 120 h. Compared to control (uncoated cover slips), cell adherence on N1, N2 and N3 was to the order of 60, 50 and 40 %, respectively, at 24 h (Fig. 1). All the composites supported cell growth as evident by MTT values at 120 h. The MTT values of the composites and control had very less difference. The growth was best in N3 (2×), followed by N2 (1.5×) and least in N1 (1.3×).

**Fig. 2:**
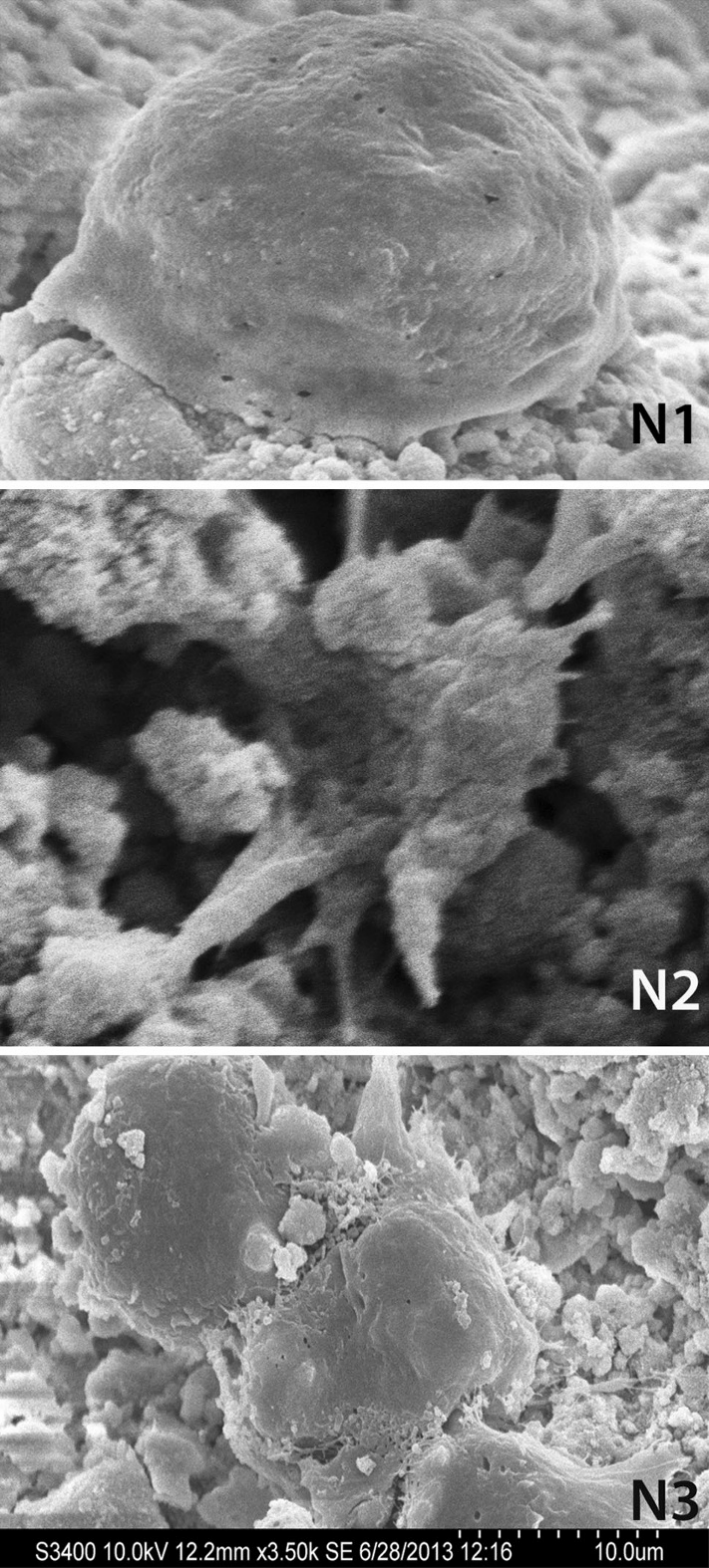
Scanning electron micrographs of cells on the nanohydroxyapatite pellets. Mesenchymal stem cells were co-cultured with nanohydroxyapatite pellets made with three different materials; the cells adhere to the pellets with in 12 h. Cells adhering on N1 (**a**); N2 (**b**) and cells on N3 composite (**c**)

The pellets composed of three composites were cultured in culture bottles with the mesenchymal stem cells derived from placenta and maintained in Wheaton’s roller cultures at 5 rpm. The pellets were removed at 6 and 12 h to investigate cell adherence. Adherence and spreading of cells over the surface and in crevices or macropores of the premoulded pellets was seen in all the three ceramic pellets. By 6–12 h, cells had not completely spread but were adhering at the site (Fig. 2). MSC spread on the nanoHA pellets did not show extensive filopodia spreading around as seen in osteoblast cell lines plated on CPC cements as reported Dalby et al. 2002. Among the three composites, N1 showed slightly more cell adherence on the surface.

Cells grown on the preformed pellets or the coated surfaces were also used for analysis of osteogenic gene expression. We used BMP2, osteocalcin and bone sialoprotein as markers. BMP2 in particular induce osteogenic commitment of mesenchymal cells inhibiting their differentiation along the myo or adipogenic lineages (Katagiri et al. 1994). Osteocalcin is one of the few osteoblast specific genes with abundant expression in bone, with an important role in the differentiation of osteoblast progenitor cells, showing significant up-regulation observed in both matrix synthesis and mineralization (Lian et al. 1989). BSP, a hydroxyapatite-binding protein is mitogenic for pre-osteoblast cells and can promote their differentiation into mature osteoblasts, ultimately stimulating bone mineralization (Zhou et al. 1995).

**Fig. 3:**
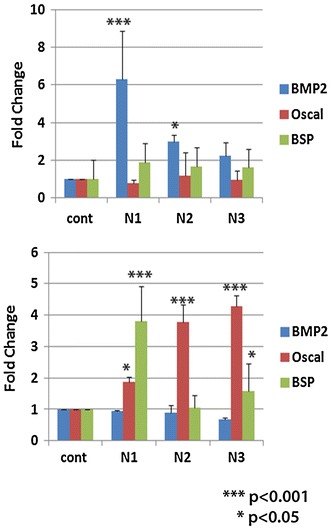
Differential gene expression of mesenchymal cells on biocomposite materials. Changes in osteogenic gene expression in cells grown on N1, N2 and N3 after 10 (**a**) and 15 days (**b**). Real-time PCR analysis was performed for BMP2, osteocalcin and bone sialoprotein shows significant changes in gene expression

Results of the real-time PCR indicated that these composites support differentiation of the MSC, hence are osteoinductive in nature. There is significant difference, in expression of different genes. BMP2 and BSP show an initial increase in expression in all the constructs by day 10 (Fig. 3). Osteocalcin expression increased dramatically around 15 days in cells grown on all material. Of the three constructs, N1 appears to be highly osteoinductive with minimum proliferation.

In vitro assessment of any biomaterial is the first step to determine the safety and biological activity in animal models. There is no gold standard for animal testing but proof of principle can be established in small rodents. The biocompatibility and bone healing capacity of these graft material were checked in bone injury healing model developed in Wistar rats earlier (Reddy et al. 2013). Our earlier study showed that this model worked well with surgical time and no unusual immune responses. A bone drill injury of about 6 mm size was created in femur of the experimental cohorts and immediately filled with paste made with nanoHAP cements. In a parallel set of animals, the material was mixed with 100,000 cells after making paste and injected at the bone drill injury site, while setting. A setting time of 5 min was allowed before suturing back various layers at the wound site.

**Fig. 4:**
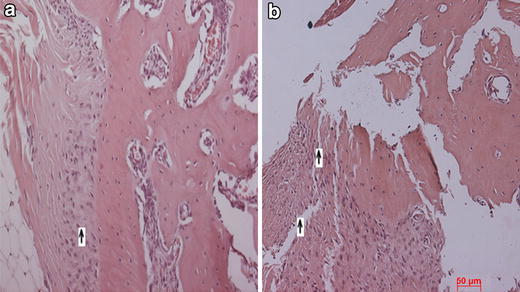
Histology of the bone at the injury site. **a** Control bone with no graft material, **b** bone with only cells at the injury site. In both cases, the bone is not completely healed and shows presence of fibrous tissue (*up arrow*) and thinning of the bone at injury site. *Scale bar* represents 50 µm

All the animals with different implants recovered well after surgery and the implantation site wound healed without any complications. The histopathological studies of the bone recovered at the end of 3 months revealed that the three groups displayed good healing at the injury site. There was no evidence of inflammation or graft rejection at the injury site. The animals without any implant or only with cells did not show complete healing of drilled hole injury. The bone had become thinner at injury site with lots of fibrous tissue around. Some sites showed complete lack of joining at the gap, Fig. 4.

**Fig. 5:**
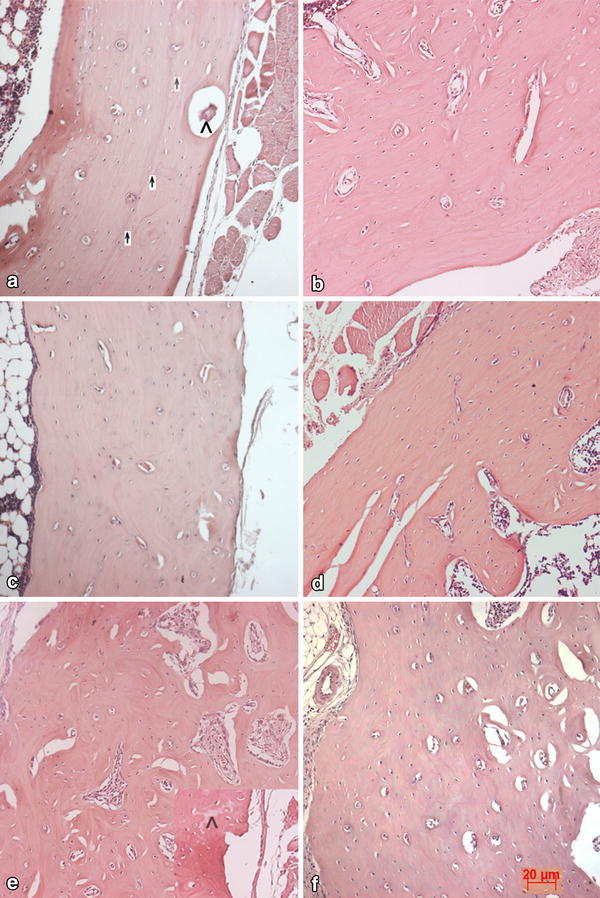
Postgraft healing of injured bone. Histology of bone with graft material at injury site displays very good healing. **a** and **b** Bone with graft material N1 **c** and **d** graft material N2 and **e** and **f** graft N3. In all the three cases, the injury appears well healed restoring the normal bone histology. **b**, **d** and **f** have the implant material along with the cells in which we find comparatively more angiogenesis. Very few sites show the remnant of material (*caps*) and very rarely the boundary of new bone formation is visible (*up arrow*). *Scale bar* represents 20 µm

The healing was much better in experimental cohort. The bone thickness was retained and the implant material was not identified in gross examination. There was no fibrous tissue formation. There were no remnants of the material traced along the length of the injury. At some sites, the boundary formation could be seen as in case of N1 (Fig. 5a). Appearance of the material was sighted very rarely (Fig. 5). The histology appeared normal with osteoblasts and vasculature surrounded by the bone matrix that appears well formed. Porosity of the cement is important for degradability and resorption. The three types of combinations used in the study appear to have integrated well at the injury site with no signs or rare traces of material. The graft material might have got resorbed and replaced by natural mineral by 12 week.

Addition of cells to the ceramic paste also appeared to work well for healing the drilled hole bone injury. The only difference noticed was the increase in angiogenesis in the histology with the three materials. Healing appeared complete as the bone structure was restored across the length at the site of injury (Fig. 5). There was no leakage of material at the wound site as the nanohydroxyapatite material could set unlike seen in our previous study (Reddy et al. 2013). The nanomaterial slurry that leaked out while filling in injury site got trapped outside the bone in the muscle and had ossified (Reddy et al. 2013). The present set of nanoparticles appeared to work better for bone healing. Hydroxyapatite being the base of this injectable graft is a natural material with osteointegrative and osteoinductive properties is so evident from this study. This particular composition has porosity and when implanted gets completely replaced by natural structure displaying good healing properties. The structure appears to be stable and maintains the right contour and volume of the bone (Fig. 5). Absence of fibrous tissue, necrotic tissue and immune cells apart from complete integration of the repaired bone with old bone makes it desirable bone filler. The other injectable graft material including cements, used for surgeries generally set with exothermic reactions and result in necrosis of surrounding tissue (Hong et al. 1991; Vallo et al. 1999). The implant site at times needs to be contoured with burr whereas simple hand contouring is sufficient. Another problem faced with the bone cements is aseptic loosening at the implant site (Fig. 5). HA added as filler to synthetic polymer-based cements or with foaming agent is also shown to be enhancing the osteointegrative properties of the cements (Dalby et al. 2002; Del Valle et al. 2007). Nanohydroxyapatite mixed with chitosan and collagen scaffolds also increases the biocompatibility and bioactivity (Peniche et al. 2010). We do not anticipate these problems of loosening as the material in present study showed good integration at implant site. Sometimes blood is reported to cause inhibition in setting of many CPC cements curtailing their use as injectables (Ishikawa et al. 1994; Miyamoto et al. 1997). We have tested this set of material by leaving the pellets in blood (Varma et al. 2012) and our present study reconfirms that setting proceeds without any problems and the material does not show any decohesion phenomenon either in culture medium or in the injury site in vivo. Decohesion of the cement can lead to inflammatory reactions (Miyamoto et al. 1999; Bohner 2000). However, no inflammatory reaction is seen in any of the combinations we have used for bone drill hole injury. In fact, in 3 months of implantation, we see a well-healed injury site with no trace of deformation, inflammation and any fibrous tissue that may indicate early integration of material.

## Conclusion

Cell adherence was good on all the composites. The composites proved to be excellent material for repair of bone drill hole injury without any adverse reactions or tissue response during the 3-month implantation trial. There was no apparent difference in the biological activity of the three graft composites, though in vitro studies showed that N1 was more pro-differentiation and N3 was more proliferating towards MSC. Further studies to ascertain these findings would be required in larger animals at different sites.

### **Authors contributions**

Sai Santosh and Snehal Raut have done the major part of the biological studies; Nitin Varma with A Sinha is responsible for synthesis of composites, Dr. Mahesh and Avinash have helped in animal studies and histology, respectively. SS has designed, analysed the study and written the manuscript.

## References

[CR1] Bai B, Jazrawi L, Kummer F, Spivak J (1999). The use of an injectable, biodegradable calcium phosphate bone substitute for the prophylactic augmentation of osteoporotic vertebrae and the management of vertebral compression fractures. Spine.

[CR2] Bohner M (2000) Calcium orthophosphates in medicine from ceramics to calcium phosphate cements. Injury. Int J Care Inj 31: S-D37–47 10.1016/s0020-1383(00)80022-4 11270080

[CR3] Brown WE, Chow LC (1983). A new calcium phosphate setting cement. J Dent Res.

[CR4] Dalby MJ, Di Silvio L, Harper EJ, Bonfield W (2002). Increasing hydroxyapatite incorporation into poly(methylmethacrylate) cement increases osteoblast adhesion and response. Biomaterials.

[CR5] Del Valle S, Mino N, Munoz F, Gonzalez A, Planell JA, Ginebra M (2007). In vivo evaluation of an injectable macroporous calcium phosphate cement. J Mater Sci Mater Med.

[CR6] Fellah BH, Weiss P, Gauthier O, Rouillon T, Pilet P, Daculsi G, Layrolle P (2006). Bone repair using a new injectable self-crosslinkable bone substitute. J Orthop Res.

[CR7] Herna´ndez L, Gurruchaga ÆM, Gon˜ ÆI (2009). Injectable acrylic bone cements for vertebroplasty based on a radiopaque hydroxyapatite. Formulation and rheological behaviour. J Mater Sci Mater Med.

[CR8] Hong YC, Wang JT, Hong CY, Brown WE, Chow LC (1991). The periapical tissue reactions to a calcium phosphate cement in the teeth of monkeys. J Biomed Mater Res.

[CR9] Hu G, Xiao L, Fu H, Bi D, Ma H, Tong P (2010). Study on injectable and degradable cement of calcium sulphate and calcium phosphate for bone repair. J Mater Sci Mater Med.

[CR10] Ishikawa K, Takagi S, Chow LC, Ishikawa Y, Eanes ED, Asaoka K (1994). Behavior of a calcium phosphate cement in simulated blood plasma in vitro. Dent Mater.

[CR11] Jackson T, Yavuzer R (2000). Hydroxyapatite cement: an alternative for craniofacial skeletal contour refinements. Br J Plast Surg.

[CR12] Katagiri T, Yamaguchi A, Komaki M, Abe E, Takahashi N, Ikeda T, Rosen V, Wozney JM, Fujisawa-Sehara A, Suda T (1994). Bone morphogenetic protein-2 converts the differentiation pathway of C2C12 myoblasts into the osteoblast lineage. J Cell Biol.

[CR13] Kokubo T (2008). Bioceramics and their clinical applications.

[CR14] Kurashina K, Kurita H, Hirano M, Kotani A, Klein CP, de Groot K (1997). In vivo study of calcium phosphate cements: implantation of an alpha-tricalcium phosphate/dicalcium phosphate dibasic/tetracalcium phosphate monoxide cement paste. Biomaterials.

[CR15] Le Geros RZ, Chohayeb A, Shulman A (1982). Apatitic calcium phosphates: possible dental restorative material. J Dent Res.

[CR16] Lian J, Stewart C, Puchacz E, Mackowiak S, Shalhoub V, Collart D, Zambetti G, Stein G (1989). Structure of the rat osteocalcin gene and regulation of vitamin D-dependent expression. Proc Natl Acad Sci USA.

[CR17] Miyamoto Y, Ishikawa K, Fukao H, Sawada M, Nagayama M, Ron M, Asaoka K (1995). In vivo setting behaviour of fast-setting calcium phosphate cement. Biomaterials.

[CR18] Miyamoto Y, Ishikawa K, Takechi M, Toh T, Yoshida Y, Nagayama M, Kon M, Asaoka K (1997). Tissue response to fast-setting calcium phosphate cement in bone. J Biomed Mater Res.

[CR19] Miyamoto Y, Ishikawa K, Takechi M, Toh T, Yuasa T, Nagayama M, Suzuki K (1999). Histological and compositional evaluations of three types of calcium phosphate cements when implanted in subcutaneous tissue immediately after mixing. J Biomed Mater Res Appl Biomater.

[CR20] Nandi SK, Roy S, Mukherjee P, Kundu B, De DK, Basu D (2006). Orthopaedic applications of bone graft and graft substitutes: a review. Am J Biochem Biotechnol.

[CR21] Oh S, Oh N, Appleford M, Ong JL (2006). Bioceramics for tissue engineering applications—a review. Am J Biochem Biotechnol.

[CR22] Peniche C, Solís Y, Davidenko N, García R (2010). Chitosan/hydroxyapatite-based composites. Biotecnología Aplicada.

[CR23] Reddy S, Wasnik S, Guha A, Kumar JM, Sinha A, Singh S (2013). Evaluation of nano-biphasic calcium phosphate ceramics for bone tissue engineering applications: in vitro and preliminary in vivo studies. J Biomater Appl.

[CR24] Schlickewei W, Schlickewei C (2006) The use of bone substitutes in the treatment of bone defects, biomaterials in regenerative medicine. In: Nadolny AJ (ed) Proceedings of the international conference, Vienna, 22–25 October 2006

[CR25] Sinha A, Nayar S, Agarwal A, Bhattacharya DP, Ramachandrarao J (2003). Synthesis of nanosized and microporous precipitated hydroxyapatite in synthetic and biopolymers. J Am Ceram Soc.

[CR26] Ślósarczyk A, Czechowska J, Paszkiewicz Z, Zima A (2010). New bone implant material with calcium sulfate and Ti modified hydroxyapatite. J Achiev Mater Manuf Eng.

[CR27] Tomoaia G, Tomoaia-Cotise M, Pop LB, Pop A, Horovitz O, Mocanu A, Jumate N, Bobos LD (2011). Synthesis and characterization of some composites based on nanostructured phosphates, collagen and chitosan. Rev Roum Chim.

[CR28] Vallo CI, Montemartini PE, Fanovich MA, Lo´pez JMP, Cuadrado TR (1999). Polymethylmethacrylate-based bone cement modified with hydroxyapatite. J Biomed Mater Res (Appl Biomater).

[CR29] Varma NP, Garai S, Sinha A (2012). Synthesis of injectable and cohesive nano hydroxyapatite scaffolds. J Mater Sci Mater Med.

[CR30] Verma NP, Sinha A (2013). Effect of solid to liquid ratio on the physical properties of injectable nanohydroxyapatite.

[CR31] Verret DJ, YadrankoDucic MD, Oxford L, Smith MD (2005). Hydroxyapatite cement in craniofacial reconstruction. Otolaryngol Head Neck Surg.

[CR32] Zhang Y, Hockin H, Xu K, Takagi S, Chow LC (2006). In-situ hardening hydroxyapatite-based scaffold for bone repair. J Mater Sci Mater Med.

[CR33] Zhou H-Y, Takita H, Fujisawa R, Mizuno M, Kuboki Y (1995). Stimulation by bone sialoprotein of calcification in osteoblast-like MC3T3-E1 cells. Calcif Tissue Int.

